# Understanding circadian regulation of mammalian cell function, protein homeostasis, and metabolism

**DOI:** 10.1016/j.coisb.2021.100391

**Published:** 2021-12

**Authors:** Alessandra Stangherlin, Estere Seinkmane, John S. O'Neill

**Affiliations:** MRC Laboratory of Molecular Biology, Cambridge, UK

**Keywords:** Circadian rhythm, Biological clock, Macromolecular crowding, Protein synthesis, Protein turnover, Homeostasis, Osmostasis, Ion transport, TORC, Respiratory oscillation, Metabolic cycle, Metabolism, Cellular function

## Abstract

Circadian rhythms are ∼24 h cycles of organismal and cellular activity ubiquitous to mammalian physiology. A prevailing paradigm suggests that timing information flows linearly from rhythmic transcription via protein abundance changes to drive circadian regulation of cellular function. Challenging this view, recent evidence indicates daily variation in many cellular functions arises through rhythmic post-translational regulation of protein activity. We suggest cellular circadian timing primarily functions to maintain proteome homeostasis rather than perturb it. Indeed, although relevant to timekeeping mechanism, daily rhythms of clock protein abundance may be the exception, not the rule. Informed by insights from yeast and mammalian models, we propose that optimal bioenergetic efficiency results from coupled rhythms in mammalian target of rapamycin complex activity, protein synthesis/turnover, ion transport and protein sequestration, which drive facilitatory rhythms in metabolic flux and substrate utilisation. Such daily consolidation of proteome renewal would account for many aspects of circadian cell biology whilst maintaining osmotic homeostasis.

## Highlights

• It is unlikely that most circadian cell functions arise through protein abundance rhythms.
• Much circadian cell physiology may be explained by rhythms in mammalian target of rapamycin complex activity.
• Temporal consolidation of proteome renewal enables efficient protein homeostasis.
• Metabolism and ion transport facilitate the bioenergetics of daily protein turnover.

## Graphical abstract

**Figure:**
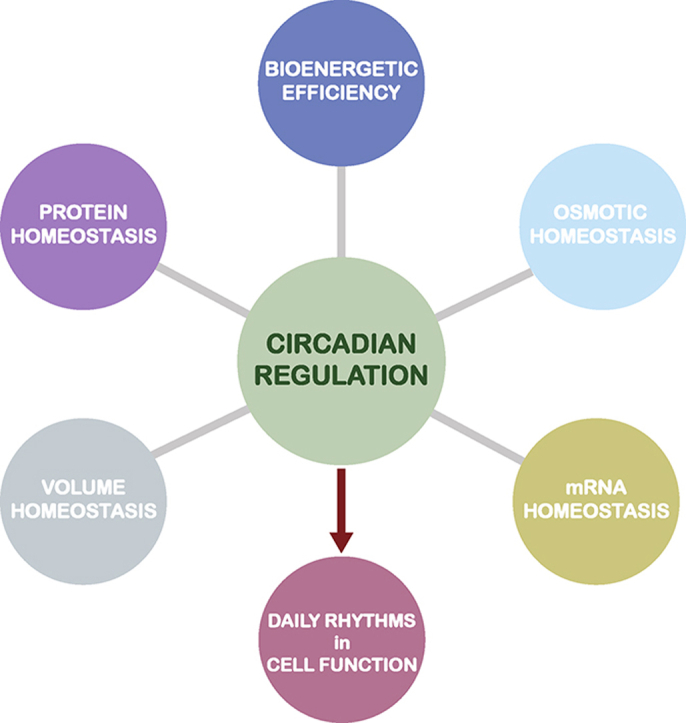

## Introduction

Daily variation is intrinsic to most aspects of mammalian physiology, with well-established clinical relevance [1], but only recently a cell-autonomous basis for understanding circadian regulation of biological function has become clear. From DNA replication to mitochondrial respiration, most cellular processes in mammals and other eukaryotes are temporally organised into ∼24 h (circadian) rhythms that are regulated by endogenous timing mechanisms. *In vivo,* rhythms are synchronised by multiple systemic cues [2,3], but their persistence in isolated cells highlights their cell-intrinsic nature.

Fibroblast motility, for example, exhibits circadian rhythms of actin polymerisation and cytoskeletal dynamics, which confer daily variation on their capacity to enact wound-healing responses, *in vitro* and *in vivo* [4]. In other primary cells, cell-intrinsic circadian regulation facilitates ∼24 h cycles of collagen deposition and turnover [5], the phagocytic activity of macrophages [6] and the electrical activity of cardiomyocytes [7]. An assumption common to these and similar studies is that temporal variation in any given biological process is directly attributable to temporal variation in the abundance of one or more proteins that directly catalyse or modulate that process.

The general hypothesis that daily variation in the abundance of specific ‘clock-controlled proteins’ is causally responsible for generating myriad cellular circadian functions is attractive and plausible. However, although several investigations provide correlational observations consistent with this model, there is little direct supporting evidence for it. Many studies have reported daily rhythms in cellular metabolism, for example, that correlate with daily abundance rhythms in the mRNA or the protein level of relevant metabolic enzymes [8]. Under physiological conditions, however, enzyme abundance almost never limits the rate of flux through metabolic pathways [9,10]. Instead, activity is regulated allosterically, by covalent modification and substrate availability [11]. It therefore seems unlikely that modest daily variation (typically <20%) in the abundance of a few metabolic enzymes has significant biological consequences [9]. Furthermore, rhythms in physiological outputs tend to have much higher amplitudes than abundance rhythms of key proteins that regulate those outputs. For example, abundance rhythms of several actin regulators identified by Hoyle et al. [4] oscillated with low amplitudes (<10%) compared with the accompanying daily variation in actin polarisation and cell motility (about 2-fold). Therefore, other mechanisms must be considered to explain how such rhythms in cellular activity emerge. We describe a model that, in principle, may allow hitherto unexplained aspects of circadian biology to be understood.

## Circadian regulation of cell function: is it all transcriptional?

*Period1/2* are immediate-early genes encoding nucleocytoplasmic proteins (Period1/2) whose activity and stability are regulated by both cell-intrinsic circadian mechanisms and extracellular signalling [2,12,13]. Period1/2 facilitate the rhythmic recruitment of promiscuous transcriptional repressors (Cryptochrome1/2) to multiple different genomic loci, including the *Period1/2* gene promoters [14, 15, 16]. Current evidence suggests changes in activity and/or abundance of ‘clock proteins,’ such as Period1/2, function as central vectors of circadian timing information that communicate daily variations in the cellular state to the nucleus [17], where they facilitate changes in chromatin architecture and transcription [2,15,18]. From this model, it is frequently assumed that resultant circadian oscillations in ‘clock-controlled genes’ generate rhythmic abundance of mRNAs and their encoded proteins, which elicit commensurate rhythms in protein function [19]. As detailed in the following, however, several recent reports are not concordant with this assumption.

The advent of readily available and mutilplexable omics technologies has expedited temporal analysis of thousands of transcripts and proteins. A direct comparison between the two has revealed poor overlap between rhythmic transcripts and rhythmic proteins. About half or, in some cases, most proteins with detectable ∼24 h rhythms do not have correspondingly rhythmic transcripts [20]. Moreover, in cases where both a protein and its transcript are rhythmic, their phases of expression often differ by 6 h or more [6,21, 22, 23, 24]. In macrophages, most rhythmic proteins with commensurately rhythmic mRNAs actually peak in advance of their mRNA [6]. Furthermore, with the exception of established circadian transcriptional regulators such as Period1/2, little similarity is seen in the identity and phase profiles of rhythmically abundant proteins when data from independent investigations are compared [22, 23, 24, 25]. This discrepancy could be explained by differences in methodology or proteomics platforms. However, if changes in protein abundance genuinely drive circadian physiology, one would expect robust and reproducible oscillations in the key clock-controlled proteins proposed to underpin such rhythmic activities within a given cellular or tissue context; but, this is rarely observed. Moreover, although most rhythmic transcripts across multiple tissues can change by 2- to 4-fold over the daily cycle [19], the amplitude of protein abundance rhythms is much lower (typically <20%). Even under diurnal conditions in the liver, where circadian rhythms are additionally synchronised and amplified by feeding and other systemic cues, 97% of rhythmically abundant proteins vary by < 2-fold [22]. Thus, excepting short-lived proteins with low baseline expression, such as Period1/2, where small variations in expression/stability may be amplified by stochastic effects [26,27], such modest changes are unlikely to affect the activity of more stable and abundant proteins [9,10,28]. It is also noteworthy that, both in cells and *in vivo*, genetic ablation of Cryptochrome1/2, which are crucial for circadian transcriptional regulation, was associated with increased temporal variation at the protein level, not a reduction [22,29].

Circadian ribosomal profiling studies have noted that there is close correlation between changes in a transcript's abundance and its translation [25,30,31]. This has been taken as corroborating evidence that transcription-derived changes in the available mRNA pool drive changes in the identity of proteins being translated. Of those genes whose transcription is circadian-regulated, however, most show no accompanying rhythms in steady-state mRNA abundance [32,33]. When coupled with strong evidence that translational initiation and elongation protect mRNAs from degradation [34], it is equally possible that the causality is opposite, that rhythmic translation underlies many rhythms in the steady-state mRNA level. Except for the negative feedback circuit effected by Period1/2, therefore, multiple investigations now suggest that circadian transcription simply cannot be assumed to regulate cell function by generating or amplifying protein rhythms. Moreover, physiological variation in protein abundance cannot be assumed to drive changes in biological function unless experimentally validated, for example, by clamping the protein's abundance at the midpoint of its normal oscillation.

## Constraints on the temporal organisation of cell biology

Over timescales of hours, at least three factors constrain the biology of living cells and must be satisfied by any model for temporal regulation of cellular function: osmotic homeostasis (osmostasis); energy homeostasis; and protein homeostasis (proteostasis). Disruption to any of these results in cellular stress responses to counteract perturbation and cell death if these responses are unsuccessful [35, 36, 37, 38, 39]. It is intuitive that cells must be in osmotic equilibrium with their extracellular environment and should not expend more biochemical resources than are available, both acutely and in the longer term. Proteostasis (the dynamic regulation of a balanced and functional proteome) is costly because protein synthesis is the most energetically expensive process that most cells undertake [40], requiring high rates of ATP and amino acid consumption that must be balanced by supply.

All cellular proteins accrue damage and/or lose function over time and so must be replaced for cells to maintain proteostasis and viability. A protein's steady-state abundance is determined by its relative rate of synthesis and degradation, which also determine its rate of renewal. Over time, any mismatch between its rate of synthesis and degradation leads to a change in abundance. The total concentration of proteins in the cell is very high (∼350 mg/mL) [41,42] and cannot change by much without disrupting osmotic and volume homeostasis. For nonproliferating cells, overall rates of protein production must be approximately matched by removal rates (via autophagy and the ubiquitin-proteasome system) for proteostasis to be maintained over timescales of hours. The replacement of macromolecular protein complexes, comprising ∼1/3 of all mammalian proteins [43], is particularly challenging as subunits must be expressed in roughly similar stoichiometries, without exceeding chaperone capacity, and orphan subunits must be wastefully degraded or else misfold and aggregate [44]. Even in primary cells, up to 50% of certain ribosomal and proteasomal subunits are degraded immediately after translation owing to their super-stoichiometric expression [44]. In consequence, cells curtail most protein synthesis until sufficient resources are available to sustain the high translation rates required for efficient protein complex assembly. This process is primarily regulated by the ubiquitous and essential target of rapamycin complexes (TORCs) [45,46], whose appropriate regulation is critical for proteostasis [47].

## Circadian regulation of target of rapamycin complex, protein synthesis and turnover

Mammalian TORC1 (mTORC1) is the master regulator of cellular anabolism versus catabolism. It integrates many different intracellular indicators of energetic and biosynthetic resources with extracellular signals to control the switch between protein synthesis and autophagic degradation, for example, by phosphorylation of ribosomal S6 kinase and autophagy-related protein 13 (ATG13) [45,48]. Similarly regulated TORC2 coordinates metabolism, actin dynamics and survival/proliferation [46]. mTORC is under circadian regulation in cultured cells and *in vivo* [7,49,50], with increased activity at biological times equivalent to the active phase *in vivo* (daytime for humans, night-time in mice) [7,51, 52, 53]. *In vivo*, daily feeding-driven (insulin pathway-dependent) stimulation of mTORC is well-characterised [2]. The cell-autonomous mechanisms driving circadian mTORC activity are not well understood, however, although regulation by daily cycles of Mg^2+^ availability and interactions with clock proteins have been proposed [51,54,55].

Daily mTORC activity rhythms stimulate daily rhythms in the protein synthesis rate [51,53,56, 57, 58]. Indeed, the translation of ribosomal subunits and ribosome biogenesis itself is also circadian-regulated [25,30,31,52,53,59,60]. The observed high amplitude rhythms in cellular protein synthesis must be powered by commensurate variation in the ATP production/consumption rate [51,61], facilitated by rhythms in glycolytic flux [62] and mitochondrial respiration [63, 64, 65]. Circadian rhythms in steady-state amino acid levels are similarly observed, consistent with daily variation in their rates of production and consumption *via* autophagy and translation, respectively [61,66].

Irrespective of biological context, however, the overall abundance of most cellular proteins does not change over the circadian cycle. This is unsurprising, considering the half-lives of most proteins are substantially longer than a day [67,68], which buffers the proteome against transcriptome fluctuations. Logically, therefore, short half-life proteins (<6 h) with constant abundance and rhythmic synthesis must be subject to a matched rhythm in degradation. Similarly, those proteins with constant synthesis and rhythmic abundance must be subject to circadian degradation [69]. Consistent with this, experimental evidence for circadian regulation of proteasomal activity and autophagy has been reported [70,71], occurring even in the absence of circadian gene expression cycles [72]. Specific regulatory mechanisms are poorly understood at this time, although rhythmic mTORC inactivity likely contributes to daily autophagy rhythms, and proteasomal activity probably varies in step with the translation rate [73,74].

Thus, both protein synthesis and degradation vary during the circadian cycle and likely depend on rhythmic mTORC activity, whereas overall changes in protein abundance are small. The parsimonious consequence would be that the combined action of daily rhythms in global protein synthesis and degradation rates lead to circadian modulation of protein turnover, which is essential for proteome renewal and therefore proteostasis [37]. Supporting this, cells and mice with genetically disrupted circadian timing exhibit impaired proteostasis [29,54]. Physiologically, the rhythmic regulation of protein synthesis and degradation would allow a portion of the proteome to be replaced each day whilst minimising changes in overall protein abundance/composition and especially beneficial with respect to multimeric protein complexes, given their high embodied energy cost [44].

## Circadian regulation of sequestration, ion transport and macromolecular crowding

The cytosol is a highly concentrated colloidal solution, with very little free solvent [41,42]. In nonproliferating cells, a proportion of mRNA transcripts, inactive/stalled ribosomes, proteasomes and other elements of the translational machinery are sequestered into nonmembrane-bound macromolecular condensates [75, 76, 77]. Circadian regulation has been reported for such phase-separated domains, for example, processing bodies and stress granules [30,78]. Activation of mTORC1 by growth factor signalling [45,46,48] and also over the circadian cycle [7,49] apparently triggers release of macromolecules from condensates and translocation from other cellular compartments [79], which increase cytosolic protein concentration by 20–30% without changing overall cellular protein levels [7,29]. The associated increase in colloidal osmotic potential is compensated by net export of K^+^, the major cellular osmolyte, whose cytosolic concentration is estimated to oscillate in antiphase with soluble protein by as much as 40 mM [7]. This osmotic compensation is partly mediated by members of the solute carrier family 12A (SLC12A) family of electroneutral cation-coupled chloride transporters, and thus lower amplitude rhythms in cellular Cl^−^ and Na^+^ are also observed. mTORC-dependent daily variation in cellular ion content provides a mechanistic basis for understanding cell-autonomous rhythms in the electrical activity of cardiomyocytes [7].

When mTORC activity is highest, the associated liberation of sequestered proteins and RNA increases cytosolic macromolecular crowding that is sufficient to reduce macromolecular diffusion rates by 15–30% [7]. As the concentration of macromolecules increases, so does the favourability of macromolecular interactions [42]. Crowding also affects biochemical reaction rates, although in a more complex way, by increasing their thermodynamic favourability while concomitantly reducing the kinetic favourability of those that are diffusion-limited [42]. Thus, mTORC-mediated circadian increases in cytosolic macromolecular crowding are expected to alter many reaction rates whilst stabilising associations between macromolecules. For example, cytoskeletal dynamics are very sensitive to crowding effects, favouring increased polymerisation rate and F-actin formation [80,81]. In principle then, circadian variation in cytosolic macromolecular crowding is sufficient to account for observed rhythms in actin dynamics [4]. Thus, TORC inhibition is predicted to abolish daily rhythms in cell migration and adhesion.

## Lessons from yeast

**Figure 1:**
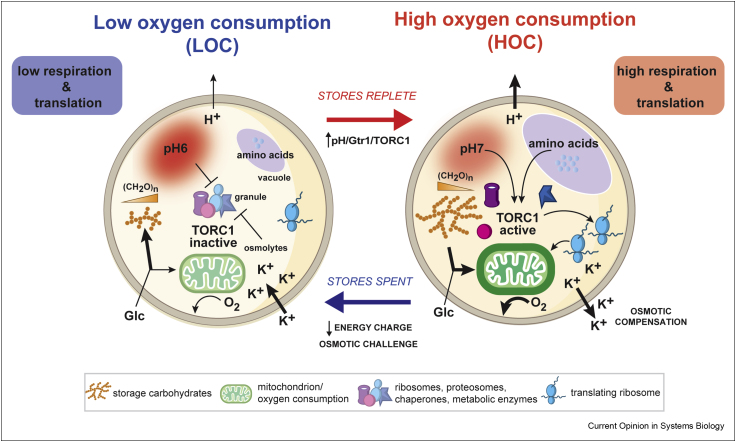
**Mechanismsof the yeast respiratory oscillation.** YROs result from temporal segregation of high rates of protein synthesis that demand high oxygen consumption (HOC) from other growth processes with low oxygen consumption (LOC). YRO, yeast respiratory oscillation; Glc, glucose; Gtr1, TORC1-stimulating GTPase.

A model that adequately satisfies the osmotic, energetic and proteostatic constraints on temporal organisation of cell biology arises from recent work in yeast [82,83] (Figure 1). Under nutrient-limited conditions, *S. cerevisiae* spontaneously undergoes ultradian (<daily) cycles of respiration, known as yeast respiratory oscillations (YROs). Each cycle lasts a few hours, characterised by a phase of low oxygen consumption (LOC), and then high oxygen consumption (HOC) that is coincident with a burst of protein synthesis. Increased respiration in HOC occurs to satisfy the energetic requirements for *de novo* protein synthesis, which is inhibited until cells have stored sufficient carbohydrates and amino acids to provide the necessary metabolic and biosynthetic substrates. The switch from LOC to HOC is triggered by intracellular acidification, TORC activation and liberation of ribosomes, proteasomes, chaperones and glycolytic enzymes from macromolecular condensates, which supply the machinery needed to sustain nascent polypeptide production. Liberation of macromolecules from condensates during HOC, which would otherwise pose an intracellular osmotic challenge, is compensated by export of K^+^ and other organic osmolytes. So important is osmostasis that the early exhaustion of stored osmolytes leads to premature termination of HOC and protein synthesis. Yeast mutants that lack YROs, because they cannot store glycogen, exhibit 2-fold increase in irreversible protein aggregation, indicating a failure of proteostasis and its intimate relationship with energy homeostasis.

Although there are clear differences between YROs and mammalian cellular circadian rhythms [84], copious similarities between the two merit some consideration [85]. YROs are similarly cell-autonomous, with oscillatory periods regulated by the same conserved kinases as circadian rhythms (casein kinase 1 and glycogen synthase kinase 3) [85]. Both entail rhythms in primary metabolism and steady-state metabolite levels [61,62,64,66] and involve cycles of macromolecular crowding, sequestration and translation rate that are largely attributable to rhythmic TORC activity (∼24 h cycles in mammalian cells, <<24 h cycles in yeast). Furthermore, both oscillations show the same rhythm in osmotic compensation, where net K^+^ export facilitates release of cytosolic proteins from other cellular compartments, with minimal change in cell volume or overall protein abundance [7]. Moreover, both exhibit extensive rhythmic regulation of the transcriptome and proteome, but, in each case, very few proteins oscillate with consistent and biologically significant amplitudes [82,83]. In the YRO, it is increasingly clear that rhythmic protein activity is driven post-translationally and facilitated by rhythms in metabolic flux, where rhythms in steady-state metabolite levels and protein activity are the key drivers of oscillations in transcription, not the other way around [83]. There is no reason this could not also be true for circadian regulation of mammalian cell biology.

Ultimately, YROs result from a ‘store then spend’ temporal compartmentalisation strategy that balances the high bioenergetic cost of efficient translation against cell growth when nutrients are sporadically available. Here, dynamic regulation of ion transport and metabolic plasticity is required to maintain osmotic and protein homeostasis during proteome remodelling. In yeast, therefore, bioenergetic constraints favour temporal organisation that promotes oscillations whose frequency varies with nutrient availability. In mammals, the oscillatory frequency is always ∼24 h, and metabolic resources are additionally balanced at the organismal scale. Indeed, nutrient intake itself usually occurs with a predictable daily rhythm that coincides with insulin pathway-dependent activation of mTORC and protein synthesis in many different cell types [2]. Cell-autonomous daily rhythms in mTORC activity and its consequences may therefore occur in anticipation of expected nutrient intake and be amplified by rhythms in insulin/IGF-1 signalling. Because mammalian cells share the same fundamental cell biology with yeast, it seems plausible that both types of oscillation evolved from a common ancestral mechanism that organises cell physiology in the most efficient manner so that temporal partitioning of metabolic resources minimises the energetic cost of macromolecular complex assembly and proteome renewal.

## Circadian control and cellular homeostasis: a model for rhythmic cell function

**Figure 2:**
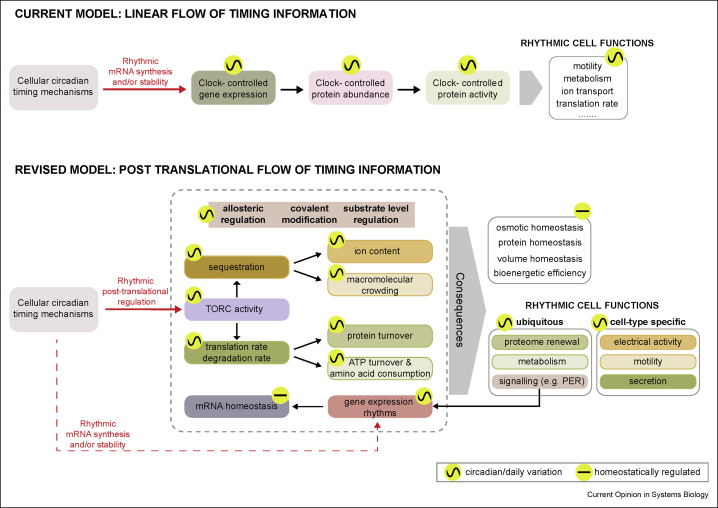
**Models for circadian regulation of cellular function.** In the traditional view, circadian gene regulation drives activity rhythms of the encoded protein to generate rhythms in various cell functions. In our revised model, many rhythmic cellular functions arise as direct or indirect consequences of daily rhythms in TORC activity. This facilitates cellular homeostasis whilst conferring circadian regulation on various general and cell-type-specific, aspects of mammalian cell biology. Box colours indicate processes that are likely causally linked, for example, rhythms in the translation rate generate rhythmic protein secretion. TORC, target of rapamycin complex.

Given the limitations faced by current models for circadian regulation of cell function (Figure 2, upper panel) outlined previously, we consider what refinements might enable their reconciliation with more recent experimental observations. We have described how several rhythmic cellular functions may simply result from the direct or indirect consequences of mTORC-mediated daily rhythms in the global translation rate and associated changes in cytosolic protein levels, crowding, metabolism and ion transport. Returning to our initial examples, phagocytosis and motility are reliant on actin dynamics, which are mTORC2-regulated and accelerated by crowding [80,81], flux through the secretory pathway is sensitive to global translation rate [86], and rhythms in cardiomyocyte electrical activity result from osmotic buffering of cytosolic protein levels [7]. Only in the final case was the mTORC dependence of rhythmic cell function tested, with modest TORC inhibition abolishing daily firing rate oscillations in cultured cardiomyocytes, as well as circadian variation in heart rate *ex vivo* and *in vivo* (under autonomic blockade).

From our current understanding then, mTORC apparently functions as part of a molecular switch between two different cellular states that impact on many different biological activities. Cell-autonomous circadian cycles of mTORC activity and related processes may therefore be sufficient to account for many different physiological rhythms that are intrinsic to mammalian cell biology (Figure 2, lower panel). This organisation of cell function allows cell volume, osmotic balance and the abundance of most proteins to be maintained at their homeostatic level and provides the most bioenergetically efficient means for the production and replacement of macromolecular complexes. It also provides a testable basis for understanding the circadian regulation of many other essential cellular processes, such as signal transduction, proteome renewal and metabolism, more broadly. Clearly, mTORC cannot directly and single-handedly regulate all of circadian cellular physiology; however, the wealth of tools developed for the manipulation of this signal transduction network allows its direct and indirect contribution to other cell-type-specific circadian functions to be readily investigated [48]. For example, mTORC-dependent changes in ion content may contribute to circadian and sleep/wake-dependent changes in the excitability of neurons.

The starting point for this model is daily mTORC activity rhythms, an output from the cell-autonomous circadian clock mechanism, by one or more modes that remain to be fully characterised [50,51,55]. mTORC activity is already known to be regulated by more than a dozen physiologically relevant intracellular cues, in addition to daily changes in growth factor signalling that also regulates its activity *in vivo* [48,87,88]. Clearly then, some combination of allosteric regulation [55], covalent modification [89] and substrate availability [51] must be sufficient to confer daily rhythms on mTORC activity, and this should be a focus of future studies. To this end, we anticipate that mass spectrometry-based investigations of the circadian mTORC-interactome and related phosphoproteome will provide valuable insight [29,89, 90, 91]. Our model solely addresses cell-autonomous circadian regulation of cell function in nonproliferating cells. It should be further validated *ex vivo* before considering the complexity introduced by cell growth/division and the many different systemic cues that cells encounter each day *in vivo,* where daily mTORC activity rhythms are also observed.

## Conclusions

Daily rhythms of gene expression and protein abundance are inadequate to explain many cellular functions that exhibit cell-autonomous circadian regulation. We suggest that daily cycles of mTORC activity and its cellular consequences are sufficient to account for large swathes of circadian physiology and prerequisite for many aspects of cellular and organismal homeostasis. We anticipate circadian cellular functions such as proteome renewal, as well as specific metabolic and signalling activities, will be common to all cells with mTORC rhythms, whereas other consequences of mTORC rhythmicity will inevitably vary with cellular context. Of course, more research is needed before we can adopt the hypothesis that circadian timing ultimately functions to temporally organise resource usage whilst allowing homeostatic regulation of essential cellular processes. If its predictions are validated, this may invite consideration of whether circadian rhythms in many cellular functions are genuinely advantageous or, in the absence of strong negative selection, were simply unavoidable from an evolutionarily perspective.

## Conflict of interest statement

Nothing declared.
